# The Role of Home-Based Exercise in Managing Common Musculoskeletal Disorders: A Narrative Review

**DOI:** 10.3390/jfmk10030326

**Published:** 2025-08-26

**Authors:** Vívian Santos Xavier Silva, Rodrigo José Battibugli Rivera, Eunice Fragoso Martins, Marco Carlos Uchida, Jean Marcos de Souza

**Affiliations:** 1 School of Physical Education, Universidade Estadual de Campinas (UNICAMP), Campinas 13083-851, Brazil; v195476@dac.unicamp.br (V.S.X.S.); uchida@unicamp.br (M.C.U.); 2 Department of Internal Medicine, School of Medical Sciences, Universidade Estadual de Campinas (UNICAMP), Campinas 13083-881, Brazil; e208215@dac.unicamp.br; 3 Independent Researcher, Campinas 13083-881, Brazil; drigorivera@gmail.com; 4 Post-Graduate Program in Medical Sciences, Universidade Estadual de Campinas (UNICAMP), Campinas 13083-887, Brazil

**Keywords:** musculoskeletal diseases, physical activity, home environment, low back pain, osteoarthritis

## Abstract

**Background**: Physical exercise can improve certain musculoskeletal conditions, but adherence remains low due to intimidating environments, limited government support, and financial constraints faced by many individuals. Home-based exercise is a potential strategy to treat that population. **Objective:** Discuss the main home-based resistance exercise protocols that have been studied and implemented for six highly prevalent musculoskeletal disorders. **Methods**: A narrative literature review was conducted, using the PubMed database to search for six highly prevalent musculoskeletal conditions: shoulder impingement syndrome (SIS), nonspecific low back pain (NSLBP), greater trochanteric pain syndrome (GTPS), knee osteoarthritis (OA), patellofemoral pain syndrome (PFPS), and Achilles tendinopathy (AT). The strategy included the listed pathologies and the keywords “physical exercise” or “physiotherapy”. Clinical trials, reviews, and retrospective studies from the last 30 years published in English, Portuguese, or Spanish were included. Only studies with sufficient details on the training protocols used and outcome measures were included in the analysis. **Results**: In SIS, exercise protocols have been more effective in the long term than in the short term. In PFPS and GTPS, strengthening the quadriceps and hip muscles helps reduce pain and improve function. For NSLBP, exercises like Pilates and core training demonstrate pain relief. In knee osteoarthritis, physical exercise improves pain, function, and quality of life both immediately and over time. Eccentric training promotes type I collagen formation in the tendons of patients with Achilles tendinopathy. **Conclusions**: Home-based resistance exercises studied and implemented in this review offer several general health benefits, including pain reduction, improved functionality, increased muscle strength, and enhanced motor control.

## 1. Introduction

The benefits of physical exercise for overall health have been extensively documented [1,2,3] and are routinely recommended in medical practice [2]. In recent decades, however, physical exercise has evolved from being a general health promotion recommendation to becoming a treatment tool for certain medical conditions, often ranking among the first intervention options. Numerous studies have evaluated the effectiveness of exercise for conditions such as fibromyalgia [4], mechanical low back pain [5], and osteoarthritis [6]. As research progresses, we may continue moving from generic exercise prescriptions for the general population to more disease-specific protocols tailored to each individual.

Verbal or written guidance from healthcare professionals regarding physical activity can increase patient adherence to exercise [7], but direct physical interventions are even more effective [8]. Despite the establishment of exercise centers as public policy in some countries and regions, participation remains low [9]. Some commonly cited reasons for the limited success of these initiatives include intimidating environments and inadequate supervision [9]. The situation is even more challenging for patients in lower-income countries, where government programs promoting exercise are scarce and patients often lack the resources to seek private physical therapists or exercise specialists.

Thus, home-based intervention models supported by the best available evidence could potentially improve adherence among the population. When considering science-based exercise prescription, however, while there is a vast number of papers analyzing the most efficient types of exercise for each condition, one may struggle to find well-described exercises to prescribe individually to their patients [10]. The goal of this literature review is to discuss the primary home-based resistance exercise protocols that have been studied and implemented for certain prevalent musculoskeletal disorders, where this form of exercise could offer significant treatment benefits. Whenever possible, we aimed to objectively list exercises that can be performed in an average household, with limited exercise equipment. Also, we sought to describe each exercise in detail in our Appendix A and Supplementary Files, hopefully providing guidance to health providers seeking home-based exercises for their patients.

## 2. Materials and Methods

We chose a narrative literature review as our strategy due to the broad scope of the topic. The PubMed database was used for our search, focusing on six highly prevalent musculoskeletal conditions: (1) shoulder impingement syndrome (SIS), (2) nonspecific low back pain (NSLBP), (3) greater trochanteric pain syndrome (GTPS), (4) patellofemoral pain syndrome (PFPS), (5) knee osteoarthritis (OA), and (6) Achilles tendinopathy (AT). The conditions were chosen based on the overall prevalence of musculoskeletal conditions [11], as well as our local epidemiology, specifically regarding situations in which a referral for physical rehabilitation was more likely [12]. We used the corresponding MeSH terms for each condition, along with the keywords “physical exercise” or “physical therapy”. We included clinical trials, reviews, and retrospective studies from the last 30 years, published in English, Portuguese, or Spanish. This approach, performed on 11 April 2024, yielded 13,792 results.

Initially, PubMed automation tools were used to select only full-text review articles, resulting in 2223 records. The remaining articles were reserved in a repository for future reference, if needed. Because the record list was still too long to screen, a second selection was performed using the PubMed “Best Match” tool, extracting only the first quartile of records for each musculoskeletal condition. This allowed the selection of 571 records for abstract screening. Researchers JMS and VSXS then selected studies that appeared relevant and had full-text versions available online. Duplicate, irrelevant, and case report studies were excluded, resulting in 85 studies in the final sample.

**Figure 1:**
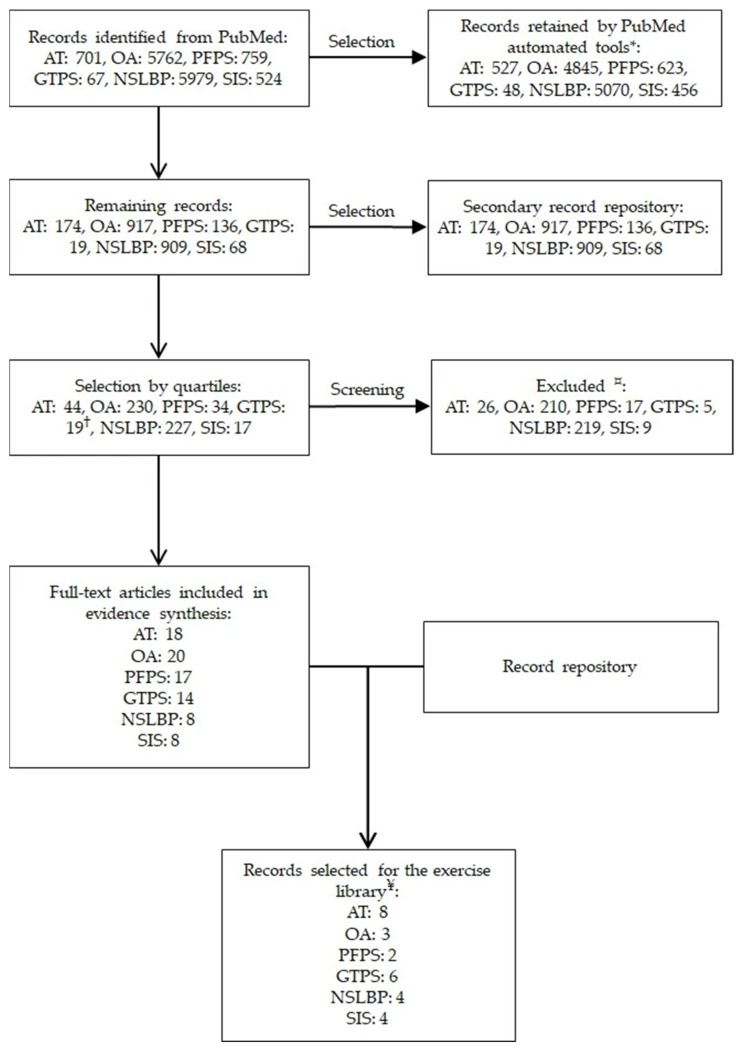
Flowchart illustrating the study search and selection protocol. * PubMed automation tools were used to select full-text reviews only. The remaining records were retained as first-level repository. † Because greater trochanteric pain syndrome’s search strategy only yielded 19 results, quartile selection was not performed. ¤ Duplicate, irrelevant, and case report studies were definitely excluded from the study. ¥ Only studies implementing fully home-based exercises with adequate descriptions were included in the exercise library. Records from the nested repository were used to compile these lists. Abbreviations: AT: Achilles tendinopathy; OA: Knee osteoarthritis; PFPS: Patellofemoral pain syndrome; GTPS: greater trochanteric pain syndrome; NSLBP: nonspecific low back pain; SIS: shoulder impingement syndrome.

The studies that passed this preliminary selection were then fully reviewed by JMS, VSXS, RBR, and EFM. For the evidence synthesis, preference was given to systematic reviews and meta-analyses. For the exercise library, only studies providing sufficient detail on the training protocols and outcome measures were included, with preference given to clinical trials. A focus group composed of the same researchers met periodically to discuss the proposed exercises to ensure that the recommendations were feasible in a typical home environment in Brazil. The results of these discussions were compiled into the manuscript. A flowchart summarizing our methodology is provided in Figure 1.

## 3. Results and Discussion

As highlighted previously, despite the enormous amount of evidence regarding exercise as therapy for musculoskeletal conditions, finding specific home-based exercises was not easy. In particular, well-described exercises for NSLBP, the most prevalent musculoskeletal condition worldwide [11], were especially difficult to find, as noted by Pieri and colleagues [10]. Overall, however, we were able to synthesize a comprehensive list of well-described exercises for all the proposed conditions, which can be found within each subsection and in Appendix A and Supplementary Files, where instructions for each exercise are provided along with video footage. In Table 1, we also highlight the results of the clinical trials in which the exercises were implemented.

### 3.1. Shoulder Impingement Syndrome (SIS)

For patients with SIS, rehabilitation is the preferred treatment over surgery [27,28]. A systematic review by the Cochrane group summarizes various exercise protocols used in studies on rotator cuff diseases [29]. Overall, a meta-analysis was not possible due to heterogeneity in the data, but in the highest-quality trial, exercise was not superior to placebo in the short term; however, it showed better outcomes over time, suggesting that its effects are more long-term [13]. Ellenbecker and Cools also described a home-based exercise protocol specifically for patients with rotator cuff diseases [30]. Since a significant percentage of patients with SIS also present with tendinopathy [31], part of the rehabilitation protocol for both conditions overlaps, allowing for some extrapolation of exercises. Finally, Clausen and colleagues [14] outlined a more intense protocol specifically for SIS. Although the clinical trial for this protocol did not show differences compared with conventional treatment, both groups demonstrated improvements, and the training routine can be used as a progression for more advanced stages of rehabilitation.

In summary, while the evidence for resisted shoulder exercises in the treatment of SIS does not strongly favor them, they do not cause significant adverse effects [29] and may provide long-term benefits [13]. Additionally, given the overall health benefits of exercise, we believe it can be considered an adjunct therapy for pain management.

Below are examples of exercises mentioned in the studies discussed above that we believe can be performed in a home-based setting [13,14,29,30,32]:

- Progressive seated shoulder press, starting from a lying position with gradual changes in the angle of inclination until seated;
- Pendulum exercises;
- Wall push-ups;
- Wall slide exercises;
- Scapular plane elevation with extended arms and supinated hands;
- Internal rotation in a side-lying position;
- External rotation in a side-lying position;
- Standing scapular protraction–retraction;
- Abduction in a side-lying position;
- Prone shoulder extension;
- External rotation (both with the arm supported and free) in the scapular plane;
- Abduction in the scapular plane;
- Prone scapular retraction.

Regarding exercise volume, Ellenbecker and Cools suggest three sets of 15 to 20 repetitions [30], and most studies in the systematic review by Page and colleagues used two to three sessions per week [29]. However, due to the low intensity of these exercises, we believe that daily sessions are feasible.

### 3.2. Patellofemoral Pain Syndrome (PFPS)

PFPS is a common cause of knee pain, accounting for approximately 5% of anterior knee pain in patients under the age of 60 [33]. Biomechanical theories for its onset include patellar malalignment and joint overload [34]. When there is an imbalance in the tensile forces acting on the patella and the patellar tendon—whether due to relative tenomuscular dysfunctions (in the knee, feet, and/or hips) or overload—an abnormal displacement of the patella over the femoral condyle may occur, leading to inflammation [34]. However, it is important to recognize that micromolecular-level factors, such as osteometabolic activity of the patella and synovial inflammation, as well as local anatomical disorders, such as neuromas or soft tissue contractures, may also contribute to the condition, supporting a multifactorial view of the phenomenon [35,36].

The management of PFPS involves various approaches, including strengthening, stretching, biofeedback, orthotics, pharmacotherapy, and surgery [34,36,37]. In general, surgical therapy is not superior to conservative treatment [38,39]. Targeted muscle strengthening, especially of the quadriceps and hip muscles, has been widely studied [40]. Chiu and collaborators demonstrated that lower limb exercises increase knee strength and the contact area of the patellofemoral joint, reducing mechanical stress and pain [41]. Balci et al. suggest that squatting may be particularly effective [42]. Nakagawa, Fukuda, and their collaborators, in turn, reported better outcomes when quadriceps strengthening was combined with hip exercises [43,44], with possible benefits when the latter precedes the former [45].

In a recent meta-analysis evaluating pain outcomes for combined quadriceps and hip strengthening, despite substantial heterogeneity, analysis of three trials (n = 112) showed a significant reduction of 3.3 points on a 10-point scale compared with placebo, with sustained benefit at one year [40]. Furthermore, when combined hip and quadriceps strengthening was compared with isolated quadriceps strengthening, the former was superior [40]. Therefore, it is likely that combined strengthening is a more favorable strategy in the conservative management of PFPS.

Stretching may also provide benefits. Peeler and Anderson, for instance, reported pain improvement with static quadriceps stretching, although not strongly correlated with flexibility [46].

In practical terms, Greaves and collaborators developed a 6-week home-based rehabilitation program for PFPS, with specific guidelines [15]. In this trial, functionality improved following the intervention, with all participants pain-free at the end of the 6 weeks [15]. Another study, from 2016, focused specifically on runners, also provided a detailed description and progression of exercises [16]. This uncontrolled 8-week clinical trial showed improvements in pain and function, although it did not increase muscle strength [16].

In summary, the exercises from these two studies include [15,16]:

- Squat (to 90 degrees of knee flexion);
- Weighted squat (using a backpack);
- Squat with elastic band resistance for hip abduction;
- Squat with combined elastic band and external load;
- Unilateral squat (lunge);
- Supine glute bridge;
- Supine glute bridge with elastic band for hip abduction;
- Unilateral glute bridge;
- Unilateral glute bridge on an unstable surface;
- Unilateral glute bridge with elastic band and unstable surface;
- Lateral band walk;
- Seated knee extension with ankle weight;
- Side-lying hip abduction;
- Side-lying hip external rotation in flexion (clamshell) with elastic band;
- Step-up;
- Quadruped plank;
- Side plank with knee support;
- Single-leg squat;
- Plank;
- Side plank;
- Hamstring and calf stretch.

Considering training intensity, the protocol by Greaves and collaborators used a broad repetition range, from 10 to 25, with participants always aiming for 25. Resistance was provided using elastic bands, loaded backpacks, and ankle weights, corresponding to 5–25% of body weight. These were applied flexibly and individually according to perceived exertion [15]. In Esculier’s study, progressively more resistant elastic bands and increasingly higher steps were used [16], with sets of 10 to 15 repetitions or 10-second isometric holds, when applicable.

Regarding training volume, De Oliveira published a meta-analysis showing that programs with fewer sessions per week resulted in greater pain reduction [47]. It is possible that, due to the flexibility of these programs, adherence was higher in those groups. The authors therefore suggest that programs begin with 12 sets per session (e.g., 4 exercises with 3 sets each), two to three times per week.

### 3.3. Greater Trochanteric Pain Syndrome (GTPS)

Greater trochanteric pain syndrome (GTPS)—a clinical entity comprising gluteus medius tendinopathy, with or without associated trochanteric bursitis—is a common musculoskeletal disorder affecting the lateral region of the hip, with a variable prevalence ranging from 10% to 25% in developed countries [48,49].

Currently, conservative treatments for GTPS include physical modalities, exercise therapy, infiltrations, and lifestyle modifications. More specifically, these treatments may involve shockwave therapy, isolated eccentric training, slow high-intensity resistance training, corticosteroid injections, platelet-rich plasma injections, and dry needling [50]. In recent years, several meta-analyses have been published on conservative treatment approaches for GTPS [51,52,53]. However, due to limitations such as an insufficient number of relevant studies, heterogeneity in inclusion criteria, and a lack of comprehensive assessments across multiple outcomes (e.g., pain and function), the relative effectiveness of these treatments remains unclear. As such, no consensus has been reached on the ideal therapeutic approach [54]. For instance, a controlled trial by Ganderton and colleagues found that home-based, gluteal-focused exercise was no more effective than education—postural and work-related advice—combined with sham exercise [17]. These findings may suggest that poor habits are as important as gluteal strength in the development or prevention of tendinopathy.

Nevertheless, the most recent comprehensive network meta-analysis, conducted by Wang et al., reaffirmed the superiority of exercise therapy for treating GTPS. It demonstrated that exercise therapy led to the greatest reductions in pain (measured using the Numeric Rating Scale, NRS) and the most significant improvements in function (measured with the Victoria Institute of Sport Assessment–Gluteal questionnaire, VISA-G). This meta-analysis confirmed the effectiveness of exercise programs in reducing pain and enhancing functional outcomes in individuals with GTPS [54].

Based on the studies discussed, examples of home-based exercises investigated for GTPS include [17,18,19,20,55,56]:

- Piriformis/gluteal stretch;
- Supine bridge exercise (hip raise);
- Straight leg raises;
- Side-lying hip abduction with knees flexed;
- Single-leg bridge with hip abduction;
- Lateral walk with squat (right and left legs);
- Single-leg stance with contralateral hip flexion and extension;
- Wall squat;
- Iliotibial band stretch;
- Standing isometric hip abduction;
- Standing isotonic hip abduction slides;
- Lateral walk (right and left legs);
- Forward lunge (right and left legs);
- Side-lying isometric hip abduction;
- Single-leg wall squat;
- Lateral walk with squat (right and left legs);
- Unilateral bridge;
- Side-lying hip abduction (right and left legs);
- Hip adductor stretch (“butterfly stretch”);
- Trunk rotator stretch (right and left sides);
- Quadruped (all-fours) unilateral hip extension;
- Prone hip extension with 90° knee flexion;
- Transverse abdominis activation;
- Quadruped hip abduction (“fire hydrant”);

Regarding volume and intensity, the protocol proposed by Notarnicola et al. consisted of 30 min of bodyweight exercises per day, five days a week, over four weeks, totaling 20 sessions [18]. However, the number of repetitions was not reported. Clifford et al. implemented a protocol involving daily isometric and isotonic exercises, also performed five days per week, but over a 12-week period, totaling 60 sessions, with each session lasting six minutes [19]. The isometric arm included two exercises. In the first, a contraction of the gluteus medius was held for 30 s and repeated six times. In the second, which was weightbearing, the contraction was held for six seconds across 10 repetitions and three sets. The exercises in the isotonic arm were similar but performed for three sets of 10 repetitions [19]. In another study, patients were instructed to exercise every day, twice daily, for 12 weeks [17]. Exercises were performed for 5 to 15 repetitions over three or four sets [17]. Finally, the protocol by Nava et al. consisted of a motor control program carried out over eight weeks, with two weekly, in-person, individualized sessions. A total of 16 sessions were completed, each lasting 50 to 60 min. The protocol consisted of isotonic and isometric strengthening exercises focused on the hip abductor and extensor muscles, with coordination through verbal commands to improve dynamic motor control of the lower limbs [55]. Progression of the exercises occurred through elastic bands, moving from easiest to most difficult, and the addition of more challenging exercises [57].

Based on the protocols discussed, a reasonable volume of therapeutic exercise for individuals with GTPS would involve sessions performed five days per week over a period of 8 to 12 weeks, totaling approximately 60 sessions. The repetition target can be around 10 repetitions for three or four sets, aiming for approximately six minutes of time under tension per session. This training volume and intensity appear sufficient to promote pain reduction, functional improvement, and increased muscular strength in patients with GTPS.

### 3.4. Nonspecific Low Back Pain (NSLBP)

Nonspecific low back pain is a common condition—the most common among non-communicable diseases [58]—and a generally benign one, which can lead to various impairments in individuals who suffer from it, causing irregular movements that may exacerbate the pain [21]. As a consequence of these disturbances, disability has been analyzed as one of the major outcomes of the condition, being present worldwide and increasing over the past three decades [59]. Occupational activities are also affected, resulting in higher costs both in terms of medical care [59] and personal and professional issues due to work-related losses [60].

Low back pain is characterized as pain located below the last ribs and above the gluteal lines, which may or may not also affect the lower limbs [60]. Being a recurrent condition, it has several possible causes; however, the most prevalent form is nonspecific low back pain [60], meaning it has no specifically diagnosed cause and may relate to many factors.

In the search for better treatments for this condition, many researchers have focused on analyzing the relationship between physical exercise and nonspecific low back pain—the most prevalent form affecting individuals across all age groups [60]. A wide range of exercises has been described to support patients in staying active, engaging in physical activity, and returning to normal routines [61]. These include motor control exercises with low and high loads [21]; aerobic training; aquatic exercises; Pilates; resistance training; sling exercises (using slings and/or elastic bands); traditional Chinese exercises; walking; yoga [62]; and stretching, stabilization, coordination, balance, flexion and extension, and strengthening exercises [60]. Back schools have also been studied and offer a multidisciplinary approach involving education, workplace positioning, and re-education, along with physical exercises [63].

The most effective exercise model for treating chronic low back pain has not yet been clearly defined, which is why numerous studies—particularly systematic reviews—continue to explore this question. Despite the absence of a standardized approach, the various strategies mentioned above highlight potential tools that can be employed. However, many studies do not report their protocols transparently, making it difficult to reproduce the methods [10]. Details such as exercise intensity and volume are often lacking, requiring further measurement and planning before practical implementation, even when other documents are used as references.

Below are some examples of exercises utilized in trials [10,21,59,60,61]:

- Alternating Straight Leg Raises;
- Supine Lumbar Rotation Stretch (Spinal Twist Stretch);
- Double Knee-to-Chest Stretch (Hip and Knee Flexion);
- Supine Bridge with Shoulder Flexion (Arm Raises);
- Transverse Abdominis Isometric Activation (Abdominal Bracing);
- Glute Bridge Exercise;
- Isometric Hip Adduction with Pillow (Adductor Squeeze);
- Hip Abduction and Adduction with Knees Flexed (Side-Lying Hip Movements);
- Supine Isometric Foot Press Against Pillow;
- Standing Forward Trunk Flexion;
- Overhead Load Lift (High Load Lift from Overhead Position);
- Supine Straight Leg Raises;
- Cat–Cow Exercise (Quadruped Lumbar Flexion and Extension);
- Isometric Forearm Plank;
- Piriformis Stretch;
- Prone Alternate Arm and Leg Lifts (Swimming Exercise, Pilates Style);
- Bird-Dog Exercise (Quadruped Contralateral Arm and Leg Reach);
- Seated Lateral Flexion Stretch (Mermaid Stretch, Pilates).

Regarding intensity, most trials utilized a moderate repetition target, equivalent to 10 RM. A reasonable volume appears to be around two to three sets of three to five exercises, performed at least twice per week. For isometric exercises, the total time under tension per set can be 30 s. Most protocols were tested over a period of 8 to 12 weeks.

### 3.5. Knee Osteoarthritis (OA)

Knee osteoarthritis (OA) is a ubiquitous condition and one of the main reasons middle-aged and elderly people seek care in primary health. It is the 11th leading cause of disability worldwide [64] and affects around 10% of men over the age of 60 [65], with the prevalence in women probably twice as high [66]. Risk factors involved in the development of OA include genetic predisposition, aging, obesity, joint malalignment, and prior joint injury or surgery [67].

The pathogenesis of primary OA is only partially understood but involves disruption in the balance between the formation and degradation of cartilage [68]. Multiple factors contribute to this disruption, including excessive inflammatory mediators and matrix-degrading enzymes [68]. The predominant degeneration that follows prompts chondrocytes to clonally expand and increase their otherwise quiescent metabolism [69]. In older individuals, mitochondrial function can be impaired, and the increasing demands for metabolic activity can elevate reactive oxygen species (ROS), leading to a redox imbalance [70,71]. The overall oxidative stress leads to chondrocyte imbalance and apoptosis [72], which, in turn, eventually results in reduced production of the cartilage matrix and, thus, of the cartilage itself.

While the mechanisms described above account for the reduction in cartilage thickness and, therefore, in articular space, they do not explain the pain experienced by individuals with OA. Studies with volunteers undergoing arthroscopy without anesthesia have shown that cartilage is aneural and avascular, and therefore insensitive to touch or injury [73,74]. Local and central mechanisms of pain sensitization are likely involved in the manifestation of symptoms [74], as well as biodynamic factors [75].

Considering the multifactorial features of OA, it is tempting to consider physical exercise as a management option to address impaired muscle function [6]. A comprehensive Cochrane meta-analysis addressed this subject in 2015. Although the study found significant variability in the prescribed exercises, the pooled results—including aerobic and various forms of strengthening exercises—indicated that the interventions produced immediate effects on pain, physical function, and quality of life, lasting up to six months [6].

Regarding strengthening exercises in particular, the benefit was described more than two decades ago in the large FAST trial, in which resistive exercise over 18 months led to gains in physical function—measured by walking, lifting objects, and entering and exiting a car—knee pain—measured by the Knee Pain Scale [76]—and physical disability [77]. A recent network meta-analysis involving 2646 patients confirmed these findings: resistance training was superior to control in alleviating knee pain—measured by the Western Ontario and McMaster Universities Arthritis Index (WOMAC) pain domain—although it was not associated with improvements in the other dimensions of the WOMAC score [78].

As for home-based exercises, a meta-analysis by Si and collaborators, pooling results from 1442 patients, found that the home-based exercise arm improved significantly more than the control in both pain and function, as measured by the WOMAC index [79]. Although the meta-analysis found a significant bias related to blinding—since it is difficult to blind a home-based intervention—these results suggest that home-based exercises are a promising strategy to address knee OA [79].

Regarding the arsenal of home-based exercises, the following list provides some examples used in previous trials [22,23,24]:

- Seated knee extension (with cuffs or resistance bands);
- Knee extension with foam roll under the knee;
- Sit-to-stand;
- Step-ups;
- Forward touchdowns from a step;
- Partial wall squats;
- Side leg raises in standing;
- Crab walk with resistance band;
- Wall push while standing on the study leg;
- Bench knee curls (with cuffs or bands);
- Heel raises;
- Squats;
- Lunges;
- Glute bridge;
- Isometric quadriceps contractions;
- Lying straight-leg lifts;
- Prone leg lifts;
- Hip adductor isometric contraction;
- Tandem walk;
- Walking with dorsiflexed and plantarflexed ankle.

Regarding volume and intensity, most studies applied protocols lasting 6 to 12 weeks [22,23,24]. Session durations ranged from 20 to 60 min, three to five times per week [6,22,23,24]. The intensity was usually sufficient to achieve a 10-repetition maximum (RM) across 1 to 3 sets [6,23,24]. Therefore, a suggested exercise prescription for knee OA could consist of 12 weeks, with three sessions per week. Three sets of 3 to 5 exercises could be performed at a moderate intensity (around 10 RM), with 60 s of rest between sets. If isometric exercises are included, the time under tension per exercise can be around 50 s per set.

### 3.6. Achilles Tendinopathy (AT)

As inherent walkers, humans depend profoundly on the gastrocnemius and soleus muscles. The confluence of these two muscles forms a thick and resistant tendon—the Achilles tendon—which inserts into the calcaneus bone [80]. This structure is highly tensile and can reliably support around 10 times the body weight during running and 4 times during walking [81,82], making it the strongest tendon in the human body [83]. Nevertheless, it is prone to injury, accounting for 20% of all tendinopathies [84].

Tendinopathy occurs when matrix formation and organization become unbalanced relative to degradation [84]. Histologically, it is characterized by collagen fiber degeneration and disorganization, increased production of type III collagen and glycosaminoglycans, and decreased vascular ingrowth [85]. Nevertheless, tendinopathies generally do not exhibit overt inflammation [80].

Mechanisms leading to Achilles tendinopathy (AT) are multifactorial and relate to both intrinsic and extrinsic factors [84]. Intrinsic factors include age—older individuals [86]—gender—male [86,87]—tendon hypovascularity, and biomechanical abnormalities (e.g., forefoot varus and pes cavus) [80,84]. Examples of extrinsic factors include sudden increases in training, poor running technique, inappropriate footwear, or training on slippery surfaces [80,87,88].

Tackling the pain in AT is challenging because the mechanisms leading to symptoms are poorly understood. Inflammation is probably not the main pathological issue, as inflammatory infiltrate is not a hallmark of the condition [80,84]. On the other hand, the degree of tendon degeneration is not directly correlated with the amount of pain, so a purely structural explanation is also insufficient [89]. Some authors propose that leakage of extracellular matrix compounds (e.g., chondroitin sulfate) and lactate produced due to impaired tendon function may act as signaling agents, promoting the overproduction of pain mediators such as substance P and glutamate [89,90]. Therefore, measures to mitigate tendon dysfunction—such as physical exercise—should be central to the management of AT.

It has been demonstrated that eccentric exercise induces type I collagen formation in the tendons of patients with AT [91]. Accordingly, more homogeneous evidence regarding this type of training has emerged in recent years, making the body of evidence on AT rehabilitation less heterogeneous than that for the other pathologies discussed in this article. One of the most well-known eccentric high-load protocols for AT is the Alfredson protocol [90], discussed in detail further. In a meta-analysis by Wilson and collaborators, of the 22 articles included, the majority of the 19 trials investigating eccentric training used the Alfredson protocol [92].

Even before the publication of Alfredson’s protocol, however, Stanish and collaborators used a somewhat similar protocol, albeit with lower volume and including stretching and icing [93]. A clinical trial comparing the two interventions found that while both improved pain and function—measured by the Victorian Institute of Sport Assessment—Achilles (VISA-A)—Alfredson’s protocol led to greater improvement [26]. On the other hand, a 2014 study evaluating alternatives to the Alfredson protocol compared the classical approach to a modified one, in which the number of repetitions was limited by pain (as opposed to the “train through pain” directive of the high-volume protocol), and found no difference in VISA-A or visual analog pain scale outcomes [25]. In fact, a later meta-analysis also found no difference between Alfredson’s protocol and other heavy-load eccentric protocols with lower volume regarding pain outcomes [92].

While the relative homogeneity of rehabilitation strategies is beneficial for healthcare providers and patients, the monotony of the Alfredson protocol can limit adherence. To provide insights into the objective progression of exercises in AT rehabilitation, Baxter et al. analyzed 30 exercises for their kinetic and kinematic properties [94]. The authors created a composite loading index, allowing the exercises to be ranked according to the load imposed on the Achilles tendon. This enables healthcare professionals to offer a broader range of exercises adapted to the patient’s stage of recovery. However, it is worth noting that the hierarchy proposed by Baxter’s study has not yet been tested in clinical trials and should be applied with caution in clinical practice.

**Figure 2:**
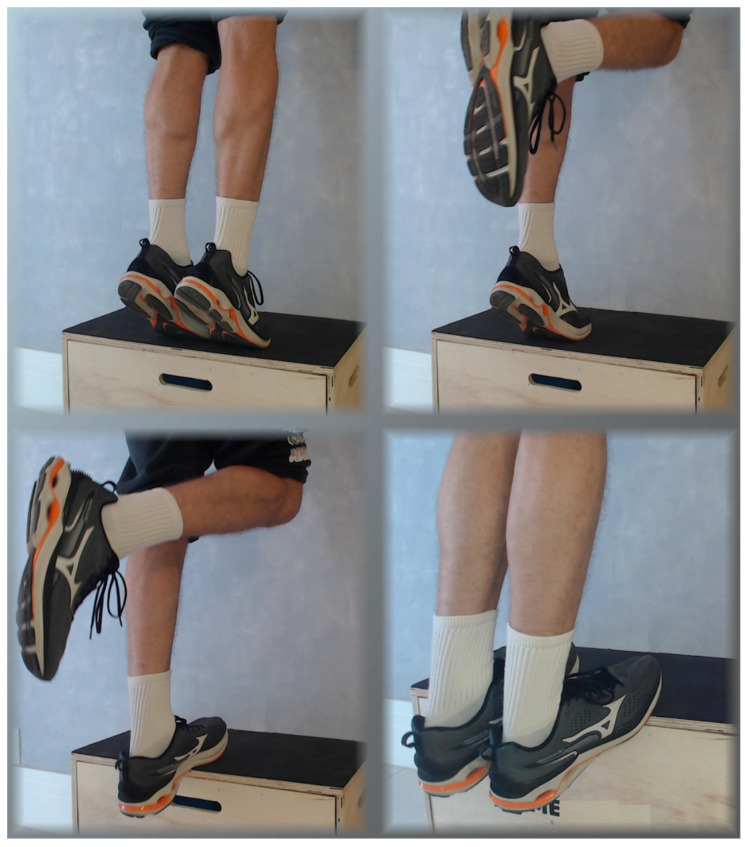
The photographs illustrate the progression of a unilateral eccentric heel drop exercise, recommended for mid-portion Achilles tendinopathy. Standing on a step or jump box, the individual uses both legs—or a handrail—to rise into maximal bilateral plantar flexion. Once at the top, the non-working leg is lifted, and the working leg slowly lowers the body in a controlled eccentric motion until reaching maximal dorsiflexion. The non-working leg then assists by joining the working leg to help lift the body back to the starting position. This ensures the exercise emphasizes the eccentric (lowering) phase. The second exercise in the protocol follows the same pattern but is performed with the knees slightly bent.

The Alfredson protocol (Figure 2): The protocol was developed and initially tested for mid-portion tendinopathy, where the disease—and consequently the point of maximal pain—is located in the middle of the tendon, approximately 2 to 6 cm proximal to its insertion at the calcaneus. Although some clinical trials evaluated the Alfredson protocol in unspecified AT (i.e., including both mid-portion and insertional forms), we suggest using the modified Alfredson protocol [95] for patients with insertional AT. In the original protocol, patients are instructed to perform 3 sets of 15 repetitions for two exercises—totaling 180 daily repetitions—twice daily, 7 days a week for 12 weeks. The first exercise is a unilateral eccentric heel drop from a step. No concentric phase should be performed—the healthy leg, the wall, or a handrail should be used to return to the starting position. The second exercise is similar but is performed with knees slightly bent, around 30 to 45 degrees. If the exercises are not sufficiently challenging, a backpack with weights should be used to bring the load to approximately 15 RM. Pain is expected and should not preclude training unless deemed disabling [90].

**Figure 3:**
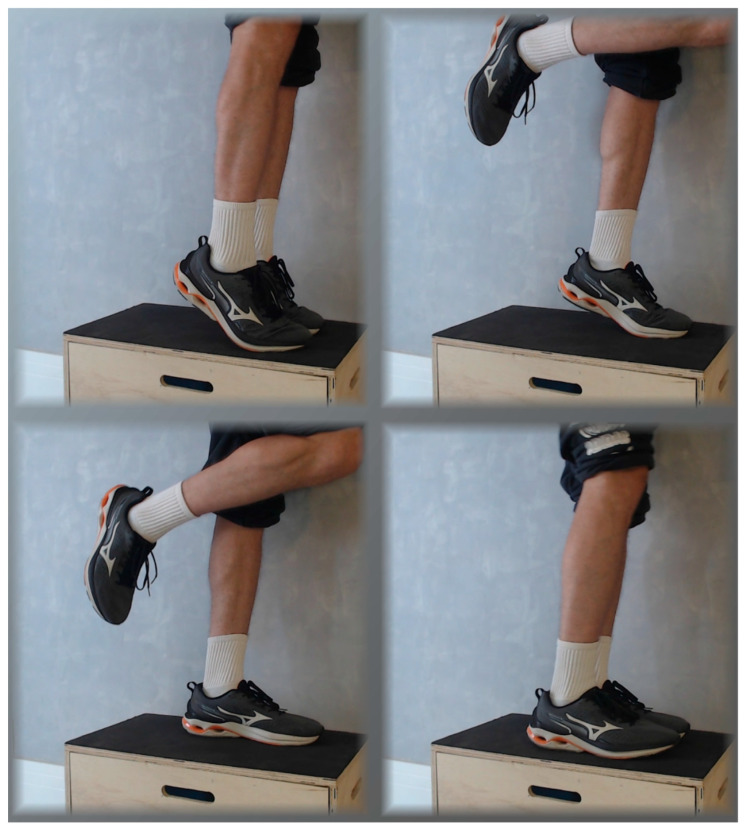
The photographs depict the progressions for a partial unilateral eccentric heel drop performed on the floor, suggested for insertional Achilles tendinopathy. The limitation of the dorsiflexion angle imposed by the floor prevents maximal stretching of the tendon. The individual uses both legs to lift into bilateral maximal plantar flexion, placing more effort on the healthy leg. From there, the rest leg is suspended and the working leg slowly drops in an eccentric motion until reaching the floor. Then, the rest leg once again joins the working leg to lift the whole body back to maximal plantar flexion, starting the exercise again. The second exercise of the protocol is performed in exactly the same way, but with the knees slightly bent.

The modified Alfredson protocol (Figure 3): For insertional AT, a slight modification was made to the protocol to avoid loading during dorsiflexion [95]. The exercises are very similar, as are the intensity and volume. However, patients are instructed to perform the exercises on the floor rather than on a step. A slight lateral shift in the center of gravity towards the supporting leg is required during the concentric phase to position the affected leg for the eccentric movement. This modification limits the range of motion and avoids load during dorsiflexion.

Some additional examples from Baxter’s study [94] include, in ascending order of loading index:

- Seated heel raise;
- Step-up;
- Lunges;
- Jumping;
- Hopping.

## 4. Conclusions

Herein, we aimed to summarize and aggregate the current knowledge regarding the rehabilitation of six highly prevalent musculoskeletal conditions through home-based workouts. We listed the main exercises used in clinical trials and provided detailed instructions, along with video guides, in Appendix A and Supplementary Materials. We conclude that the home-based resistance exercises studied here offer several general health benefits, including pain reduction, improved functionality, increased muscle strength, and enhanced motor control. Most exercises can be performed successfully with minimal equipment, such as elastic bands or ankle weights. When even these are unavailable, items like a loaded backpack, a chair, or a staircase can serve as substitutes, making these routines adaptable to most households. The ability to exercise at home may be a game-changer in physical rehabilitation, and future studies exploring these approaches are likely to be well received.

## Figures and Tables

**Table 1:** Summary of findings of the clinical trials utilized for the exercise library.

Condition	First Author, Year	N	Intervention	Control	Outcomes of Interest	Results	Observations
SIS	Bennell, 2010 [13]	120	Manual therapy (10w) plus home exercises (12w)	“Sham” therapy, no exercise	SPADI	NS	
					NRS	Favors active	
					Global rate	Favors active	
					SF36	Favors active	
SIS	Clausen, 2021 [14]	200	Home-based high-volume training, 15w	Standard care	SPADI	NS	All better within groups
					Shoulder strength	NS	
					NRS	NS	
PFPS	Greaves, 2021 [15]	16	Home-based resistance training, 6w	N/A	NRS	NS	
					KOOS	↑	
					AKPS	↑	
					Quadriceps strength	NS	
					TSK	↑	
PFPS	Esculier, 2016 [16]	21	Home-based resistance training, 8w	N/A	KOS-ADLS	↑	
					VAS-U	↑	
					VAS-W	↑	
					VAS-R	↑	
					Strength	NS	
GTPS	Ganderton, 2018 [17]	94	Home-based resistance training, 12w	Sham-exercise	VISA-G	NS	All better within groups. A subanalysis of responders showed the active arm responded better
					OHS	NS	
					AQoL	NS	
					HOOS	NS	
GTPS	Notarnicola, 2023 [18]	44	Home-based resistance training, 4w	ESWT, crossover trial	NRS	NS	All better within groups
					LEFS	NS	
					RMS	NS	
GTPS	Clifford, 2019 [19]	30	Home-based isometric training, 12w	Home-based isotonic training, 12w	VISA-G	NS	Effect size better for isotonic, but no difference within or between groups
					NRS	NS	
					GRC	NS	
					PCS	NS	
					HOOS	NS	Pain and QoL domains of HOOS within group difference in isotonic
					EuroQoL 5D-5L	NS	
GTPS	Marcioli, 2024 [20]	26	Bodyweight training, hips and core, 4w	Bodyweight training, hips only, 4w	VAS	NS	Improvements within groups for VISA and VAS
					VISA-G	NS	
					PBT	NS	
					SBT	↑	
NSLBP	Aasa, 2015 [21]	70	Bodyweight resistance training, 8w	High-load, gym-based training, 8w	PSFS	↑	↑PSFS sustained for 12mo
					VAS	NS	
					PPB	Mostly NS	All better within groups
OA	Bennell, 2017 [22]	148	Home-based resistance training, 3mo	Education only	NRS	↑	
					WOMAC	↑	
					AQoL-2	↑	
OA	Chen, 2019 [23]	141	Home-based resistance training, 12w	Education only	WOMAC	↑	
					STS	↑	
					TUG	↑	
					AIMS2-SF	↑	
OA	Kuru Çolak, 2017 [24]	78	Home-based resistance training, 6w	PT	VAS	PT better	
					6MWT	NS	
					Quadriceps strength	PT better	
AT	Stevens, 2014 [25]	28	Alfredson protocol, “do as tolerated” volume, 6w	Alfredson protocol, standard, 6w	VISA-A	NS	
					VAS	NS	
AT	Stasinopoulos, 2013 [26]	41	Alfredson’s protocol	Stanish’s protocol	VISA-A	Superiority of Alfredson’s protocol	Both improved

↑ Refers to improvement in the respective scale. 6MWT: Six-minute Walking Test; AIMS2-SF: Short form of the Arthritis Impact Measurement Scales 2; AKPS: Kujala Anterior Knee Pain Scale; AQoL: Assessment of Quality of Life; AT: Achilles tendinopathy; ESWT: Extracorporeal Shockwave Therapy; GRC: Global rating of change; GTPS: Greater trochanteric pain syndrome; HOOS: Hip Disability and Osteoarthritis Outcome Score; KOOS: Knee Injury and Osteoarthritis Outcome Score; KOS-ADLS: Knee Outcome Survey—Activities of Daily Living Scale; LEFS: Lower Extremity Functional Scale; NRS: Numeric rating scale; NS: non-significant; NSLBP: Nonspecific low back pain; OA: Knee osteoarthritis; OHS: Oxford Hip Score; PBT: Prone Bridge Test; PCS: Pain Catastrophizing Scale: PFPS: Patellofemoral pain syndrome; PPB: Physical Performance Battery; PSFS: Patient-Specific Functional Scale; PT: Physiotherapy; QoL: Quality of life; RMS: Roles and Maudslay Score; SBT: Supine Bridge Test; SF36: 36-item Short Form Health Survey; SIS: Shoulder impingement syndrome; SPADI: Shoulder Pain and Disability Index; STS: Sit to stand test; TSK: Tampa Scale for Kinesiophobia; TUG: Timed up-and-go test; VAS-R: Visual analogue scale—Running pain; VAS-U: Visual analogue scale—Usual pain; VAS-W: Visual analogue scale—Worst pain; VISA-A: Victorian Institute of Sports Assessment—Achilles tendinopathy; VISA-G: Victorian Institute of Sports Assessment—Gluteal Tendinopathy; WOMAC: Western Ontario McMaster Universities Osteoarthritis Index.

## Data Availability

All gathered data are available in this manuscript.

## Supplementary Materials

The following supporting information can be downloaded at https://www.mdpi.com/article/10.3390/jfmk10030326/s1, Video S1: SIS, Video S2: PFPS, Video S3: GTPS, Video S4: NSLBP, Video S5: OA.

## Appendix A

This appendix accompanies the manuscript “The Role of Home-Based Exercise in Managing Common Musculoskeletal Disorders: A Narrative Review.” It provides detailed explanations of the execution of exercises for each musculoskeletal condition described in the manuscript—except for the Achilles tendinopathy exercises, which are already described within the manuscript with pictures. Each description also includes an explanatory video, available in the Supplementary Materials.

Regarding the video content:

(1) All data were created and filmed by the authors for educational purposes only.
(2) Filming was intentionally conducted in household environments, keeping furniture, decorations, and objects, to best represent a typical Brazilian home.
(3) Some footage displays the word “HoBIT”, which stands for “Home-based Individualized Training” and represents a non-profit project by the researchers at Universidade Estadual de Campinas, aimed at disseminating home-based training for low-income populations.

### Appendix A.1. Shoulder Impingement Syndrome

- Progressive Seated Shoulder Press: Start lying flat, palms facing forward. Press until arms are fully extended and slowly lower back to the starting position. Gradually increase the bench angle (e.g., 30°, 45°, 60°) until seated upright.
- Pendulum Exercises (Codman’s Pendulum): Lean forward, supporting yourself with one hand on a table. Let the other arm hang loosely, relaxed. Gently swing the arm in small circles (clockwise/counterclockwise) or side-to-side motions. Use momentum, not muscle force.
- Wall Push-Ups: Stand facing a wall, feet shoulder-width apart. Place hands on the wall slightly wider than shoulders. Bend elbows to bring chest toward the wall, then push back to start. Keep the body straight (avoid sagging hips). Adjust difficulty by stepping closer (easier) or farther (harder) from the wall.
- Wall Slide Exercises: Stand facing the wall, arms bent at 90°. Slowly slide arms upward, using your fingers for support. Return to the starting position.
- Scapular Plane Elevation with Extended Arms and Supinated Hands: Stand with arms extended at 30–45° from the body (scapular plane), palms up. Raise arms to shoulder height (or higher), then lower slowly.
- Internal Rotation in Side-Lying Position: Lie on your side, elbow bent at 90°, forearm resting on the bed. Rotate the arm inward toward the ceiling.
- External Rotation in Side-Lying Position: Lie on your side, elbow bent at 90°, forearm across the abdomen. Rotate the arm upward (like opening a book).
- Standing Scapular Protraction–Retraction: Stand against a wall, with the shoulder blades touching it. Feel your scapulae opening and closing as they slide on the wall. Focus on moving the shoulder blades, not the arms.
- Abduction in Side-Lying Position: Lie on your side, arm resting along the body. Lift the arm upward to 45° (or higher), then lower.
- Prone Shoulder Extension: Lie face down on the bed, arms resting close to the body. Lift the arm backward (extend at the shoulder), just above the torso.
- External Rotation in Scapular Plane (Supported/Free): Place elbow on the back of a chair, at 90° abduction, rotate forearm up. The free version is the same, but the arm is unsupported.
- Abduction in Scapular Plane: Raise the arms diagonally from the thighs to the opposite shoulder, keeping them extended. The movement simulates drawing a sword, with the palms facing upward at the top.
- Prone Scapular Retraction: Lie face down, arms close to the sides. Squeeze shoulder blades together while lifting the shoulders at torso height. Lead with the scapulae, not the arms.

### Appendix A.2. Patellofemoral Pain Syndrome

- Squat (to 90 Degrees of Knee Flexion): Stand with feet shoulder-width apart, toes slightly turned out. Lower hips back and down as if sitting in a chair, bending knees up to 90 degrees. Keep chest up, knees aligned with toes. Push through heels to return to standing.
- Weighted Squat (Using a Backpack): Same exercise. Wear a loaded backpack securely on your shoulders.
- Squat with Elastic Band Resistance for Hip Abduction: Place a resistance band just above knees. Perform a squat while actively pressing knees outward against the band. If you do not have an elastic band, wrap a rope or shoelace above the knees.
- Squat with Combined Elastic Band and External Load: Wear a backpack and resistance band above knees.
- Unilateral Squat (Lunge): Step one foot forward, lower until both knees are at 90 degrees. The back knee hovers above the floor. Push through front heel to return.
- Supine Glute Bridge: Lie on your back, knees bent, feet flat. Lift hips until shoulders to knees form a straight line. Squeeze glutes at the top, then lower slowly.
- Supine Glute Bridge with Elastic Band for Hip Abduction: Place a band above knees. Perform a glute bridge while pressing knees outward. If you do not have an elastic band, wrap a rope or shoelace above the knees.
- Unilateral Glute Bridge: Lift one foot off the ground, extending the leg. Push through the grounded heel to lift hips.
- Unilateral Glute Bridge on Unstable Surface: Place the grounded foot on a pillow, foam pad, or Bosu ball. Perform a single-leg bridge.
- Unilateral Glute Bridge with Elastic Band + Unstable Surface: Combine single-leg bridge, unstable surface, and band above knees.
- Lateral Band Walk: Place a band around ankles or above knees. Take small sideways steps, maintaining tension. It can be performed without the band.
- Seated Knee Extension with Ankle Weight: Sit on a chair, ankle weight attached. Slowly extend one leg until straight, then lower. A bean or sugar sack (1Kg) can be used for resistance.
- Side-Lying Hip Abduction: Lie on one side, legs stacked. Lift top leg upward, then lower. Do not let the torso roll backward.
- Side-Lying Hip External Rotation (Clamshell): Lie on side, knees bent 90°. You can wrap a band above knees or not. Keep feet together, lift top knee like a clamshell opening.
- Step-Up: Step one foot onto a bench/box, push through heel to stand tall. Lower slowly back down.
- Quadruped Plank: The easy progression of the plank. On a quadruped position, supported by knees and elbows, lean forward while maintain the abdomen flat and hips flexed. Hold for the duration specified.
- Side Plank with Knee Support: Lie on one side, prop up on elbow with knees bent. Lift hips to form a straight line from knees to shoulders.
- Single-Leg Squat: Stand on one leg, lower hips back and down (as far as control allows). Use a chair for regression or hold onto support.
- Plank: Hold a push-up position on elbows/toes, body straight. Engage glutes and core to prevent sagging. Open scapulae and hold for the duration specified.
- Side Plank: Lie on one side, prop up on elbow/forearm. Lift hips until body forms a straight line. Do not let hips drop forward or backward.
- Hamstring and Calf Stretch: Stand in a staggered stance, press back heel into the floor.

### Appendix A.3. Greater Trochanteric Pain Syndrome

- Piriformis/gluteal Stretch: Lie on your back with knees bent and feet flat on the floor. Cross your right ankle over your left thigh, just above the knee, forming a “4-shape” position. Gently pull your left thigh toward your chest until you feel a stretch in the right hip and buttock. Keep your back flat on the floor and hold the position for the specified duration. Repeat on the other side.
- Supine Bridge (Hip Raise): Lie on your back, knees bent, feet flat. Lift hips until shoulders to knees form a straight line. Squeeze glutes at the top, then lower slowly.
- Straight Leg Raises: Lie on your back and lift one straight leg until you can, then lower slowly.
- Side-Lying Hip Abduction with Knees Flexed: Lie on your side with knees bent at 90° and feet together. Lift the top leg while keeping the knees flexed, then lower it with control.
- Single-Leg Bridge with Hip Abduction: Perform a glute bridge with one leg extended straight. At the top, press the working leg’s knee outward against a band. If you do not have an elastic band, wrap a rope or shoelace above the knees.
- Lateral Walk with Squat: Squat slightly, then step sideways (right, then left).
- Single-Leg Stance with Contralateral Hip Flexion/Extension: Stand on one leg. Swing the opposite leg forward (flexion), then backward (extension).
- Wall Squat: Stand with your back against a wall, feet shoulder-width apart. Lower your hips back and down as if sitting in a chair, bending the knees to about 90°. Keep your chest up and knees aligned with your toes. Push through your heels to return to standing. The wall makes the exercise easier than the free version. A ball or broom handle can be used to slide along the wall during the movement.
- Iliotibial Band Stretch: Cross one leg behind the other and lean toward the front leg’s side. Hold for time.
- Standing Isometric Hip Abduction: Stand while holding the back of a chair for support, keeping your legs straight. Move one leg outward as far as possible and hold the top position for the specified duration.
- Standing Isotonic Hip Abduction Slides: Same movement as above, but performed multiple times for repetitions.
- Lateral Walk (Right/Left Legs): Place a band around ankles or above knees. Take small sideways steps, maintaining tension. It can be performed without the band.
- Forward Lunge (Right/Left Legs): Step one foot forward, lower until both knees are at 90 degrees. The back knee hovers above the floor. Push through front heel to return.
- Side-Lying Isometric Hip Abduction: Lie on your side, top leg slightly lifted. Hold the position for the specified time.
- Single-Leg Wall Squat: Stand with your back against a wall, feet shoulder-width apart, and lift one foot off the ground. Lower your hips back and down as if sitting in a chair, bending the supporting knee up to 90°. Push through your heel to return to standing. The wall makes the exercise easier than the free version. A ball or broom handle can be used to slide along the wall during the movement.
- Unilateral Bridge: Lie on your back, knees bent, feet flat. Lift one foot off the ground, extending the leg. Push through the grounded heel to lift hips, squeeze glutes at the top, then lower slowly.
- Side-Lying Hip Abduction (Right/Left Legs): Lie on your side, lift the top leg straight up, then lower.
- Hip Adductor Stretch (Butterfly Stretch): Lie on your back with soles of feet together, knees bent outward. Let the knees open freely and hold for the specified duration.
- Trunk Rotator Stretch (Right/Left Sides): Lie on your back and open your arms. Bend the knees over your chest and rotate to the side, trying to maintain the upper back in contact with the ground. Hold for the specified duration and switch sides.
- Quadruped Unilateral Hip Extension: On hands and knees, extend one leg upward while keeping the knee bent at 90°. Imagine trying to touch the ceiling with your foot, but maintain a straight back in line with your torso.
- Prone Hip Extension with 90° Knee Flexion: Lie face down, bend one knee to 90°. Lift the thigh slightly off the ground. For an extra challenge, hold the top position for 3 s in every repetition.
- Transverse Abdominis Activation: Lie on your back, knees bent. Exhale, draw the belly button inward without moving the pelvis.
- Quadruped Hip Abduction (“Fire Hydrant”): On hands and knees, lift one knee outward (like a dog at a fire hydrant).

### Appendix A.4. Nonspecific Low Back Pain

- Alternating Straight Leg Raises: Lie on your back with legs extended. Lift one leg to 45–60°, keeping it straight. Lower slowly, then repeat with the opposite leg.
- Supine Lumbar Rotation Stretch (Spinal Twist Stretch): Lie on your back, arms out to sides. Bend one knee and rotate it across the body, keeping shoulders flat. Hold for the specified duration.
- Double Knee-to-Chest Stretch: Lie on your back and hug both knees to your chest. Hold for time.
- Supine Bridge with Shoulder Flexion (Arm Raises): Lie on your back with arms at your sides. Slowly raise your arms overhead, keeping them straight, while pressing your heels into the floor to lift your hips, aligning them with your torso.
- Transverse Abdominis Isometric Activation (Abdominal Bracing): Lie on your back, knees bent. Exhale, draw the belly button inward without moving the pelvis.
- Glute Bridge Exercise: Lie on your back, knees bent, feet flat. Lift hips until shoulders to knees form a straight line. Squeeze glutes at the top, then lower slowly.
- Isometric Hip Adduction with Pillow: Lie on your side, knees bent, place a pillow between knees. Squeeze the pillow for 5–10 s, then relax.
- Side-Lying Hip Abduction (Knees Flexed): Lie on your side with knees bent at 90° and feet together. Lift the top leg while keeping the knees flexed, then lower it with control.
- Side-Lying Hip Adduction (Knees Flexed): Lie on your side, with the bottom knee bent 90°. Lift the bottom knee toward the ceiling.
- Supine Isometric Foot Press Against Pillow: Lie on your back, place a pillow against the soles of your feet. Press feet into the pillow for 5–10 s, then relax.
- Standing Forward Trunk Flexion: Stand tall and slowly bend forward from the hips, keeping your back straight. Let your torso hinge forward until comfortable, then return to standing by extending at the hips. Go only as far as you can while maintaining a straight back.
- Overhead Load Lift: Stand with arms extended overhead (holding a light weight). Slowly lower arms to shoulder height, then raise back up.
- Supine Straight Leg Raises: Lie on your back and lift one straight leg until you can, then lower slowly.
- Cat–Cow Exercise: On hands and knees, round your back, tuck chin to chest. Then, arch back, lift head/tailbone.
- Isometric Forearm Plank: Hold a plank on forearms and toes, body straight. Engage core and glutes and hold for time.
- Piriformis Stretch: Lie on your back with knees bent and feet flat on the floor. Cross your right ankle over your left thigh, just above the knee, forming a “4-shape” position. Gently pull your left thigh toward your chest until you feel a stretch in the right hip and buttock. Keep your back flat on the floor and hold the position for the specified duration. Repeat on the other side.
- Prone Alternate Arm/Leg Lifts (Swimming Exercise): Lie face down, lift one arm and the opposite leg simultaneously. Alternate sides in a controlled motion.
- Bird-Dog Exercise: On hands and knees, extend one arm and the opposite leg. Hold briefly, then switch sides.
- Seated Lateral Flexion Stretch (Mermaid Stretch): Sit on the floor with legs folded to one side in a comfortable position. Place one hand on the floor for support and extend the opposite arm overhead. Gently lean your torso sideways toward the supporting hand, creating a stretch along the side of your body. Keep your hips grounded and hold the position for the specified duration. Repeat on the other side.

### Appendix A.5. Knee Osteoarthritis

- Seated Knee Extension (with cuffs or resistance bands): Sit tall in a chair with cuffs/bands attached just above ankles. Slowly extend one knee fully while keeping thigh on chair. A bean or sugar sack (1Kg) can alternatively be used for resistance.
- Knee Extension with Foam Roll Under Knee: Lay down and place foam roll under the working knee. Contract the quadriceps to lift the knee from the bed. Do not use your hips.
- Sit-to-Stand: Begin seated in chair with feet flat. Lean slightly forward and push through heels to stand up. Slowly sit back down with control. You can start using a broom for support.
- Step-ups: In a staircase, step up with the entire foot. Step down slowly, leading with same leg.
- Forward Touchdowns from Step: Stand on step with one foot slightly off edge. Slowly lower hanging foot to tap floor and return to start using standing leg strength.
- Partial Wall Squats: Stand with back against wall, feet forward. Slide down until knees reach 30–45° flexion. A ball or broom handle can be used to slide along the wall during the movement. As you progress, aim to lower until 90 degrees.
- Side Leg Raises in Standing: Stand tall holding chair for balance. Lift one leg directly out to side. Slowly lower with control.
- Crab Walk with Resistance Band: Place band around thighs/ankles. Assume partial squat position and take sideways steps while maintaining tension. You can also do the exercise without the band.
- Wall Push While Standing on Study Leg: Stand facing a wall with your hands placed at shoulder height. Lift the foot nearest to the wall off the floor so that you are standing on the study leg. Keeping your body upright, push gently against the wall while maintaining balance on the supporting leg. Hold for a few seconds and return.
- Bench Knee Curls (Hip Bucks): Lie on your back with your heels resting on a bench and knees bent. Keep your arms at your sides for support. Press through your heels to lift your hips until your body forms a straight line from shoulders to knees.
- Heel Raises: On a step, rise up onto toes as high as possible. Lower slowly until tolerated, generating stretch on the hamstrings at the bottom.
- Squats: Feet shoulder-width apart, toes slightly out. Lower hips back and down. Keep chest up and knees tracking toes.
- Lunges: Step one foot forward, lower until both knees are at 90 degrees. The back knee hovers above the floor. Push through front heel to return.
- Glute Bridge: Lie on your back, knees bent, and feet flat. Lift hips until shoulders to knees are straight, squeezing glutes at the top. Lower slowly with control.
- Isometric Quadriceps Contractions: Lie on your back with legs extended. Press back of knee down into surface and hold the contraction for 5–10 s. Relax and repeat.
- Lying Straight-Leg Lifts: Lie on your back and lift one straight leg until you can, then lower slowly.
- Prone Leg Lifts: Lie face down with legs straight. Lift one leg off the floor. Keep pelvis stable, avoid arching the back. Lower with control and repeat for the prescribed number of repetitions.
- Hip Adductor Isometric Contraction: Lie on your side, knees bent, place a pillow between knees. Squeeze the pillow for 5–10 s, then relax.
- Tandem Walk: Walk heel-to-toe in a straight line.
- Walking with Dorsiflexed/Plantarflexed Ankle: Walk on heels with toes lifted and on toes, heels lifted.

## References

[1] Sattelmair J., Pertman J., Ding E.L., Kohl H.W., Haskell W., Lee I.M. (2011). Dose response between physical activity and risk of coronary heart disease: A meta-analysis. Circulation.

[2] Piercy K.L., Troiano R.P., Ballard R.M., Carlson S.A., Fulton J.E., Galuska D.A., George S.M., Olson R.D. (2018). The Physical Activity Guidelines for Americans. JAMA.

[3] Jakicic J.M., Kraus W.E., Powell K.E., Campbell W.W., Janz K.F., Troiano R.P., Sprow K., Torres A., Piercy K.L. (2019). Association between Bout Duration of Physical Activity and Health: Systematic Review. Med. Sci. Sports Exerc..

[4] Busch A.J., Barber K.A., Overend T.J., Peloso P.M., Schachter C.L. (2007). Exercise for treating fibromyalgia syndrome. Cochrane Database Syst. Rev..

[5] Hayden J.A., van Tulder M.W., Malmivaara A., Koes B.W. (2005). Exercise therapy for treatment of non-specific low back pain. Cochrane Database Syst. Rev..

[6] Fransen M., McConnell S., Harmer A.R., Van der Esch M., Simic M., Bennell K.L. (2015). Exercise for osteoarthritis of the knee: A Cochrane systematic review. Br. J. Sports Med..

[7] Orrow G., Kinmonth A.L., Sanderson S., Sutton S. (2012). Effectiveness of physical activity promotion based in primary care: Systematic review and meta-analysis of randomised controlled trials. BMJ.

[8] Kettle V.E., Madigan C.D., Coombe A., Graham H., Thomas J.J.C., Chalkley A.E., Daley A.J. (2022). Effectiveness of physical activity interventions delivered or prompted by health professionals in primary care settings: Systematic review and meta-analysis of randomised controlled trials. BMJ.

[9] Williams N.H., Hendry M., France B., Lewis R., Wilkinson C. (2007). Effectiveness of exercise-referral schemes to promote physical activity in adults: Systematic review. Br. J. Gen. Pract..

[10] Pieri E., Bonetti F., Pellicciari L., Scipioni F. (2022). Well-described exercises for chronic low back pain in Life Science Literature: A systematic review. J. Back Musculoskelet. Rehabil..

[11] Storheim K., Zwart J.A. (2014). Musculoskeletal disorders and the Global Burden of Disease study. Ann. Rheum. Dis..

[12] Souza C.d.S., Oliveira A.S.d. (2015). Prevalência de encaminhamentos às doenças musculoesqueléticas segundo a classificação estatística internacional de doenças (CID-10): Reflexões para formação do fisioterapeuta na área de musculoesquelética. Fisioter. Pesqui..

[13] Bennell K., Wee E., Coburn S., Green S., Harris A., Staples M., Forbes A., Buchbinder R. (2010). Efficacy of standardised manual therapy and home exercise programme for chronic rotator cuff disease: Randomised placebo controlled trial. BMJ.

[14] Clausen M.B., Hölmich P., Rathleff M., Bandholm T., Christensen K.B., Zebis M.K., Thorborg K. (2021). Effectiveness of Adding a Large Dose of Shoulder Strengthening to Current Nonoperative Care for Subacromial Impingement: A Pragmatic, Double-Blind Randomized Controlled Trial (SExSI Trial). Am. J. Sports Med..

[15] Greaves H., Comfort P., Liu A., Lee H., Richard J. (2021). How effective is an evidence-based exercise intervention in individuals with patellofemoral pain?. Phys. Ther. Sport.

[16] Esculier J.F., Bouyer L.J., Roy J.S. (2016). The Effects of a Multimodal Rehabilitation Program on Symptoms and Ground-Reaction Forces in Runners With Patellofemoral Pain Syndrome. J. Sport Rehabil..

[17] Ganderton C., Semciw A., Cook J., Moreira E., Pizzari T. (2018). Gluteal Loading Versus Sham Exercises to Improve Pain and Dysfunction in Postmenopausal Women with Greater Trochanteric Pain Syndrome: A Randomized Controlled Trial. J. Womens Health.

[18] Notarnicola A., Ladisa I., Lanzilotta P., Bizzoca D., Covelli I., Bianchi F.P., Maccagnano G., Farì G., Moretti B. (2023). Shock Waves and Therapeutic Exercise in Greater Trochanteric Pain Syndrome: A Prospective Randomized Clinical Trial with Cross-Over. J. Pers. Med..

[19] Clifford C., Paul L., Syme G., Millar N.L. (2019). Isometric versus isotonic exercise for greater trochanteric pain syndrome: A randomised controlled pilot study. BMJ Open Sport Exerc. Med..

[20] Marcioli M.A.R., Cunha A.P.R.R.d., Macedo C.d.S.G. (2024). Effect of muscle strengthening on pain, functionality, muscle endurance and postural control in women with greater trochanter pain syndrome: A randomized clinical trial. Braz. J. Pain.

[21] Aasa B., Berglund L., Michaelson P., Aasa U. (2015). Individualized low-load motor control exercises and education versus a high-load lifting exercise and education to improve activity, pain intensity, and physical performance in patients with low back pain: A randomized controlled trial. J. Orthop. Sports Phys. Ther..

[22] Bennell K.L., Nelligan R., Dobson F., Rini C., Keefe F., Kasza J., French S., Bryant C., Dalwood A., Abbott J.H. (2017). Effectiveness of an Internet-Delivered Exercise and Pain-Coping Skills Training Intervention for Persons With Chronic Knee Pain: A Randomized Trial. Ann. Intern Med..

[23] Chen H., Zheng X., Huang H., Liu C., Wan Q., Shang S. (2019). The effects of a home-based exercise intervention on elderly patients with knee osteoarthritis: A quasi-experimental study. BMC Musculoskelet. Disord..

[24] Kuru Çolak T., Kavlak B., Aydoğdu O., Şahin E., Acar G., Demirbüken İ., Sarı Z., Çolak İ., Bulut G., Polat M.G. (2017). The effects of therapeutic exercises on pain, muscle strength, functional capacity, balance and hemodynamic parameters in knee osteoarthritis patients: A randomized controlled study of supervised versus home exercises. Rheumatol. Int..

[25] Stevens M., Tan C.W. (2014). Effectiveness of the Alfredson protocol compared with a lower repetition-volume protocol for midportion Achilles tendinopathy: A randomized controlled trial. J. Orthop. Sports Phys. Ther..

[26] Stasinopoulos D., Manias P. (2013). Comparing two eccentric exercise programmes for the management of Achilles tendinopathy. A pilot trial. J. Bodyw. Mov. Ther..

[27] Beard D.J., Rees J.L., Cook J.A., Rombach I., Cooper C., Merritt N., Shirkey B.A., Donovan J.L., Gwilym S., Savulescu J. (2018). Arthroscopic subacromial decompression for subacromial shoulder pain (CSAW): A multicentre, pragmatic, parallel group, placebo-controlled, three-group, randomised surgical trial. Lancet.

[28] Vandvik P.O., Lähdeoja T., Ardern C., Buchbinder R., Moro J., Brox J.I., Burgers J., Hao Q., Karjalainen T., van den Bekerom M. (2019). Subacromial decompression surgery for adults with shoulder pain: A clinical practice guideline. BMJ.

[29] Page M.J., Green S., McBain B., Surace S.J., Deitch J., Lyttle N., Mrocki M.A., Buchbinder R. (2016). Manual therapy and exercise for rotator cuff disease. Cochrane Database Syst. Rev..

[30] Ellenbecker T.S., Cools A. (2010). Rehabilitation of shoulder impingement syndrome and rotator cuff injuries: An evidence-based review. Br. J. Sports Med..

[31] Sasiponganan C., Dessouky R., Ashikyan O., Pezeshk P., McCrum C., Xi Y., Chhabra A. (2019). Subacromial impingement anatomy and its association with rotator cuff pathology in women: Radiograph and MRI correlation, a retrospective evaluation. Skeletal. Radiol..

[32] Codman E.A. (1990). Rupture of the supraspinatus tendon. 1911. Clin. Orthop. Relat. Res..

[33] Gaitonde D.Y., Ericksen A., Robbins R.C. (2019). Patellofemoral Pain Syndrome. Am. Fam. Physician.

[34] Rixe J.A., Glick J.E., Brady J., Olympia R.P. (2013). A review of the management of patellofemoral pain syndrome. Phys. Sportsmed..

[35] Dye S.F. (2005). The pathophysiology of patellofemoral pain: A tissue homeostasis perspective. Clin. Orthop. Relat. Res..

[36] Bolgla L.A., Boling M.C. (2011). An update for the conservative management of patellofemoral pain syndrome: A systematic review of the literature from 2000 to 2010. Int. J. Sports Phys. Ther..

[37] Fulkerson J.P. (2002). Diagnosis and treatment of patients with patellofemoral pain. Am. J. Sports Med..

[38] Bizzini M., Childs J.D., Piva S.R., Delitto A. (2003). Systematic review of the quality of randomized controlled trials for patellofemoral pain syndrome. J. Orthop. Sports Phys. Ther..

[39] Kettunen J.A., Harilainen A., Sandelin J., Schlenzka D., Hietaniemi K., Seitsalo S., Malmivaara A., Kujala U.M. (2007). Knee arthroscopy and exercise versus exercise only for chronic patellofemoral pain syndrome: A randomized controlled trial. BMC Med..

[40] Nascimento L.R., Teixeira-Salmela L.F., Souza R.B., Resende R.A. (2018). Hip and Knee Strengthening Is More Effective Than Knee Strengthening Alone for Reducing Pain and Improving Activity in Individuals With Patellofemoral Pain: A Systematic Review With Meta-analysis. J. Orthop. Sports Phys. Ther..

[41] Chiu J.K., Wong Y.M., Yung P.S., Ng G.Y. (2012). The effects of quadriceps strengthening on pain, function, and patellofemoral joint contact area in persons with patellofemoral pain. Am. J. Phys. Med. Rehabil..

[42] Balci P., Tunay V.B., Baltaci G., Atay A.O. (2009). The effects of two different closed kinetic chain exercises on muscle strength and proprioception in patients with patellofemoral pain syndrome. Acta Orthop. Traumatol. Turc..

[43] Nakagawa T.H., Muniz T.B., Baldon Rde M., Dias Maciel C., de Menezes Reiff R.B., Serrão F.V. (2008). The effect of additional strengthening of hip abductor and lateral rotator muscles in patellofemoral pain syndrome: A randomized controlled pilot study. Clin. Rehabil..

[44] Fukuda T.Y., Melo W.P., Zaffalon B.M., Rossetto F.M., Magalhães E., Bryk F.F., Martin R.L. (2012). Hip posterolateral musculature strengthening in sedentary women with patellofemoral pain syndrome: A randomized controlled clinical trial with 1-year follow-up. J. Orthop. Sports Phys. Ther..

[45] Dolak K.L., Silkman C., Medina McKeon J., Hosey R.G., Lattermann C., Uhl T.L. (2011). Hip strengthening prior to functional exercises reduces pain sooner than quadriceps strengthening in females with patellofemoral pain syndrome: A randomized clinical trial. J. Orthop. Sports Phys. Ther..

[46] Peeler J., Anderson J.E. (2007). Effectiveness of static quadriceps stretching in individuals with patellofemoral joint pain. Clin. J. Sport Med..

[47] de Oliveira N.T., Lopez P., Severo-Silveira L., Almeida G.P.L., Baroni B.M. (2023). Dose-response effect of lower limb resistance training volume on pain and function of women with patellofemoral pain: A systematic review and meta-regression. Phys. Ther. Sport.

[48] Albers I.S., Zwerver J., Diercks R.L., Dekker J.H., Van den Akker-Scheek I. (2016). Incidence and prevalence of lower extremity tendinopathy in a Dutch general practice population: A cross sectional study. BMC Musculoskelet. Disord..

[49] Williams B.S., Cohen S.P. (2009). Greater trochanteric pain syndrome: A review of anatomy, diagnosis and treatment. Anesth. Analg..

[50] Reid D. (2016). The management of greater trochanteric pain syndrome: A systematic literature review. J. Orthop..

[51] Gazendam A., Ekhtiari S., Axelrod D., Gouveia K., Gyemi L., Ayeni O., Bhandari M. (2022). Comparative Efficacy of Nonoperative Treatments for Greater Trochanteric Pain Syndrome: A Systematic Review and Network Meta-Analysis of Randomized Controlled Trials. Clin. J. Sport Med..

[52] Kjeldsen T., Hvidt K.J., Bohn M.B., Mygind-Klavsen B., Lind M., Semciw A.I., Mechlenburg I. (2024). Exercise compared to a control condition or other conservative treatment options in patients with Greater Trochanteric Pain Syndrome: A systematic review and meta-analysis of randomized controlled trials. Physiotherapy.

[53] He Y., Lin Y., He X., Li C., Lu Q., He J. (2023). The conservative management for improving Visual Analog Scale (VAS) pain scoring in greater trochanteric pain syndrome: A Bayesian analysis. BMC Musculoskelet. Disord..

[54] Wang S.Q., Guo N.Y., Liu W., Huang H.J., Xu B.B., Wang J.Q. (2025). Effect of conservative treatment on greater trochanteric pain syndrome: A systematic review and network meta-analysis of randomized controlled trials. J. Orthop. Surg. Res..

[55] Thomaz de Aquino Nava G., Baldini Prudencio C., Krasic Alaiti R., Mendes Tozim B., Mellor R., Rodrigues Pedroni C., Mércia Pascon Barbosa A., Tavella Navega M. (2022). Motor control exercises versus general exercises for greater trochanteric pain syndrome: A protocol of a randomized controlled trial. PLoS ONE.

[56] Chan M.K., Chow K.W., Lai A.Y., Mak N.K., Sze J.C., Tsang S.M. (2017). The effects of therapeutic hip exercise with abdominal core activation on recruitment of the hip muscles. BMC Musculoskelet. Disord..

[57] Picha K.J., Almaddah M.R., Barker J., Ciochetty T., Black W.S., Uhl T.L. (2019). Elastic Resistance Effectiveness on Increasing Strength of Shoulders and Hips. J. Strength Cond. Res..

[58] Owen P.J., Miller C.T., Mundell N.L., Verswijveren S., Tagliaferri S.D., Brisby H., Bowe S.J., Belavy D.L. (2020). Which specific modes of exercise training are most effective for treating low back pain? Network meta-analysis. Br. J. Sports Med..

[59] Hayden J.A., Ellis J., Ogilvie R., Malmivaara A., van Tulder M.W. (2021). Exercise therapy for chronic low back pain. Cochrane Database Syst. Rev..

[60] Lizier D.T., Perez M.V., Sakata R.K. (2012). Exercícios para tratamento de lombalgia inespecífica. Rev. Bras. Anestesiol..

[61] Cordeiro A.L.L., Oliveira A.P.S., Cerqueira N.S., Santos F.A.F., Oliveira A.M.S. (2022). Pilates method on pain in patients with low back pain: Systematic review. Braz. J. Pain.

[62] Grooten W.J.A., Boström C., Dedering Å., Halvorsen M., Kuster R.P., Nilsson-Wikmar L., Olsson C.B., Rovner G., Tseli E., Rasmussen-Barr E. (2022). Summarizing the effects of different exercise types in chronic low back pain—A systematic review of systematic reviews. BMC Musculoskelet. Disord..

[63] Parreira P., Heymans M.W., van Tulder M.W., Esmail R., Koes B.W., Poquet N., Lin C.W.C., Maher C.G. (2017). Back Schools for chronic non-specific low back pain. Cochrane Database Syst. Rev..

[64] Cross M., Smith E., Hoy D., Nolte S., Ackerman I., Fransen M., Bridgett L., Williams S., Guillemin F., Hill C.L. (2014). The global burden of hip and knee osteoarthritis: Estimates from the global burden of disease 2010 study. Ann. Rheum. Dis..

[65] Woolf A.D., Pfleger B. (2003). Burden of major musculoskeletal conditions. Bull. World Health Organ..

[66] Geng R., Li J., Yu C., Zhang C., Chen F., Chen J., Ni H., Wang J., Kang K., Wei Z. (2023). Knee osteoarthritis: Current status and research progress in treatment (Review). Exp. Ther. Med..

[67] Xia B., Di C., Zhang J., Hu S., Jin H., Tong P. (2014). Osteoarthritis pathogenesis: A review of molecular mechanisms. Calcif. Tissue Int..

[68] Lee A.S., Ellman M.B., Yan D., Kroin J.S., Cole B.J., van Wijnen A.J., Im H.J. (2013). A current review of molecular mechanisms regarding osteoarthritis and pain. Gene.

[69] Hwang H.S., Kim H.A. (2015). Chondrocyte Apoptosis in the Pathogenesis of Osteoarthritis. Int. J. Mol. Sci..

[70] Chistiakov D.A., Sobenin I.A., Revin V.V., Orekhov A.N., Bobryshev Y.V. (2014). Mitochondrial aging and age-related dysfunction of mitochondria. BioMed Res. Int..

[71] Bolduc J.A., Collins J.A., Loeser R.F. (2019). Reactive oxygen species, aging and articular cartilage homeostasis. Free Radic. Biol. Med..

[72] Yudoh K., van Trieu N., Nakamura H., Hongo-Masuko K., Kato T., Nishioka K. (2005). Potential involvement of oxidative stress in cartilage senescence and development of osteoarthritis: Oxidative stress induces chondrocyte telomere instability and downregulation of chondrocyte function. Arthritis Res. Ther..

[73] Dye S.F., Vaupel G.L., Dye C.C. (1998). Conscious neurosensory mapping of the internal structures of the human knee without intraarticular anesthesia. Am. J. Sports Med..

[74] Vincent T.L., Miller R.E. (2024). Molecular pathogenesis of OA pain: Past, present, and future. Osteoarthr. Cartil..

[75] He Y., Li Z., Alexander P.G., Ocasio-Nieves B.D., Yocum L., Lin H., Tuan R.S. (2020). Pathogenesis of Osteoarthritis: Risk Factors, Regulatory Pathways in Chondrocytes, and Experimental Models. Biology.

[76] Rejeski W.J., Ettinger W.H., Shumaker S., Heuser M.D., James P., Monu J., Burns R. (1995). The evaluation of pain in patients with knee osteoarthritis: The knee pain scale. J. Rheumatol..

[77] Ettinger W.H., Burns R., Messier S.P., Applegate W., Rejeski W.J., Morgan T., Shumaker S., Berry M.J., O’Toole M., Monu J. (1997). A randomized trial comparing aerobic exercise and resistance exercise with a health education program in older adults with knee osteoarthritis. The Fitness Arthritis and Seniors Trial (FAST). JAMA.

[78] Mo L., Jiang B., Mei T., Zhou D. (2023). Exercise Therapy for Knee Osteoarthritis: A Systematic Review and Network Meta-analysis. Orthop. J. Sports Med..

[79] Si J., Sun L., Li Z., Zhu W., Yin W., Peng L. (2023). Effectiveness of home-based exercise interventions on pain, physical function and quality of life in individuals with knee osteoarthritis: A systematic review and meta-analysis. J. Orthop. Surg. Res..

[80] Maffulli N., Sharma P., Luscombe K.L. (2004). Achilles tendinopathy: Aetiology and management. J. R. Soc. Med..

[81] Komi P.V., Fukashiro S., Järvinen M. (1992). Biomechanical loading of Achilles tendon during normal locomotion. Clin. Sports Med..

[82] Oda H., Sano K., Kunimasa Y., Komi P.V., Ishikawa M. (2017). Neuromechanical Modulation of the Achilles Tendon During Bilateral Hopping in Patients with Unilateral Achilles Tendon Rupture, Over 1 Year After Surgical Repair. Sports Med..

[83] Tarantino D., Palermi S., Sirico F., Corrado B. (2020). Achilles Tendon Rupture: Mechanisms of Injury, Principles of Rehabilitation and Return to Play. J. Funct. Morphol. Kinesiol..

[84] Tarantino D., Mottola R., Resta G., Gnasso R., Palermi S., Corrado B., Sirico F., Ruosi C., Aicale R. (2023). Achilles Tendinopathy Pathogenesis and Management: A Narrative Review. Int. J. Environ. Res. Public Health.

[85] Maffulli N., Barrass V., Ewen S.W. (2000). Light microscopic histology of achilles tendon ruptures. A comparison with unruptured tendons. Am. J. Sports Med..

[86] Maffulli N., Wong J., Almekinders L.C. (2003). Types and epidemiology of tendinopathy. Clin. Sports Med..

[87] Kvist M. (1991). Achilles tendon injuries in athletes. Ann. Chir. Gynaecol..

[88] Selvanetti A., Cipolla M., Puddu G. (1997). Overuse tendon injuries: Basic science and classification. Oper. Tech. Sports Med..

[89] Khan K.M., Cook J.L., Maffulli N., Kannus P. (2000). Where is the pain coming from in tendinopathy? It may be biochemical, not only structural, in origin. Br. J. Sports Med..

[90] Alfredson H., Pietilä T., Jonsson P., Lorentzon R. (1998). Heavy-load eccentric calf muscle training for the treatment of chronic Achilles tendinosis. Am. J. Sports Med..

[91] Langberg H., Ellingsgaard H., Madsen T., Jansson J., Magnusson S.P., Aagaard P., Kjaer M. (2007). Eccentric rehabilitation exercise increases peritendinous type I collagen synthesis in humans with Achilles tendinosis. Scand. J. Med. Sci. Sports.

[92] Wilson F., Walshe M., O’Dwyer T., Bennett K., Mockler D., Bleakley C. (2018). Exercise, orthoses and splinting for treating Achilles tendinopathy: A systematic review with meta-analysis. Br. J. Sports Med..

[93] Stanish W.D., Rubinovich R.M., Curwin S. (1986). Eccentric exercise in chronic tendinitis. Clin. Orthop. Relat Res..

[94] Baxter J.R., Corrigan P., Hullfish T.J., O’Rourke P., Silbernagel K.G. (2021). Exercise Progression to Incrementally Load the Achilles Tendon. Med. Sci. Sports Exerc..

[95] Jonsson P., Alfredson H., Sunding K., Fahlström M., Cook J. (2008). New regimen for eccentric calf-muscle training in patients with chronic insertional Achilles tendinopathy: Results of a pilot study. Br. J. Sports Med..

